# Effects of Four Nanomaterials on the Performance and Microstructure of Coal Gangue-Based Geopolymers

**DOI:** 10.3390/ma19061095

**Published:** 2026-03-12

**Authors:** Zhenhua Wang, Wei Lan, Zhiwen Jia, Tiantian Jiang, Xiqi Liu, Gang Wang, Minghua Hu

**Affiliations:** 1 School of Civil and Architecture Engineering, East China University of Technology, Nanchang 330013, China; zhh200802@163.com (Z.W.); zwjia518@163.com (Z.J.);; 2 Pearl River Water Resources Research Institute, Guangzhou 510611, China; 3 Key Laboratory of Water Security Guarantee in Guangdong-Hong Kong-Marco Greater Bay Area of Ministry of Water Resources, Foshan 528244, China; 4 State Key Laboratory of Intelligent Deep Metal Mining and Equipment, Shaoxing University, Shaoxing 312000, China

**Keywords:** coal gangue, compressive strength, geopolymer, microstructure, nanomaterials, pore structure, rheological properties

## Abstract

This study aimed to enhance the slurry performance and durability of coal gangue-based geopolymers (CGGP) by incorporating four types of nanomaterials: nano-SiO_2_ (NS), graphene oxide (GO) nanosheets, nano-CaCo_3_ (NC), and nano-Al_2_O_3_ (NA). The microstructure and underlying mechanisms were thoroughly investigated using scanning electron microscopy (SEM) and energy-dispersive X-ray spectroscopy (EDS). The results indicate that the type and dosage of nanomaterials significantly influence the rheological properties, strength development, setting time, porosity, and water absorption of CGGP. Specifically, the addition of GO nanosheets drastically reduced fluidity, with a 73.33% decrease in flowability compared to the control group at a 2.0 wt.% dosage. Nano-SiO_2_ exhibited the most pronounced effect in improving compressive strength and shortening the setting time, with the optimal accelerating effect observed at a 1.5 wt.% dosage. Nano-CaCO_3_ primarily acts as a filler. Though its reactivity is relatively low, at an appropriate dosage (1.5 wt.%), it can effectively reduce porosity and water absorption. Moreover, at a dosage of 1 wt.%, it exhibits the optimal 28-day compressive strength, which is 54.18% higher than that of the blank group. Nano-Al_2_O_3_ demonstrated a relatively stable accelerating effect on setting and yielded the best pore structure and strength at a 1.5 wt.% dosage. SEM analysis revealed that the incorporation of an appropriate amount of NC particles significantly improved the microstructural densification of the polymer. Concurrently, EDS results confirmed the positive influence of the nano-Al_2_O_3_ material on the distribution of hydration products and the interfacial structure. This research provides an important theoretical basis and technical support for the high-performance design and widespread engineering application of coal gangue-based geopolymers.

## 1. Introduction

Driven by the global construction industry’s urgent pursuit of sustainable development, the development of novel green building materials has emerged as a critical research priority. Conventional Portland cement (OPC) production, responsible for approximately 8% of global anthropogenic CO_2_ emissions [1,2], presents a significant environmental burden. This has accelerated research into geopolymers (GP) as a low-carbon alternative. Synthesized via the alkali activation of aluminosilicate precursors, geopolymer technology not only reduces CO_2_ emissions by over 70% compared to OPC—primarily through the extensive incorporation of industrial waste streams in its formulation—but also facilitates the resource recovery of solid industrial byproducts [3,4]. This dual advantage positions geopolymers as a pivotal technology for advancing sustainable construction practices.

Coal gangue, the primary solid waste generated from coal mining and washing processes, presents a massive global environmental challenge. Its annual global discharge reaches staggering levels of 1.0 to 1.5 billion tons, with China accounting for over 50% of this volume [5,6]. Stockpiling this waste not only consumes vast tracts of land but also poses significant environmental risks, including spontaneous combustion, dust emissions, and potential leaching of heavy metals. However, coal gangue possesses a significant advantage: it is rich in silica (SiO_2_, 40–60%) and alumina (Al_2_O_3_, 15–30%) [7,8,9], making it a highly suitable aluminosilicate precursor for geopolymer synthesis. Despite this compositional suitability, the inherent limitations of coal gangue—namely its relatively low reactivity and sluggish reaction kinetics—often result in geopolymers exhibiting drawbacks such as slow early-age strength development and non-uniform microstructure [5,10].

In recent years, nanomaterials have demonstrated significant potential for modifying geopolymers, leveraging their unique surface effects and small-size effects [11,12]. Research indicates that the appropriate incorporation of nano-silica (NS) can substantially enhance the mechanical properties of geopolymer concrete, primarily attributed to its pore-filling capability and ability to increase the micro-density of the gel phase [13]. Several scholars have systematically investigated NS-reinforced geopolymer composites. For instance, when NS is composited with metakaolin (MK)-based geopolymers, it promotes the formation of various unsaturated bonds and diverse hydroxyl bonding configurations [14]. Wang J et al. [15] found that incorporating NS into high-calcium fly ash-based geopolymers improved their compressive and flexural strengths under ambient curing conditions. Specifically, specimens containing 2 wt.% NS exhibited 32% and 38% higher compressive and flexural strengths, respectively, compared to the control group at 90 days of curing. The authors attributed this enhancement to the reaction between the calcium components and NS, which promoted the generation of additional polymeric gel. However, further research [16] suggests that excessive NS dosage may lead to component agglomeration, potentially resulting in a structure with lower compactness. Notably, activated NS with high surface free energy can significantly accelerate the geopolymerization reaction kinetics. Beyond nano-silica, nano-alumina (NA) has also proven effective in enhancing geopolymer performance. Yang Zhenghui et al. [17] demonstrated that NA, leveraging its exceptionally high specific surface area, exerts a pronounced nucleation effect that promotes the formation of additional hydration products (e.g., C-A-S-H gel). This significantly enhances the mechanical properties of geopolymer mortar (CGS), albeit at the expense of reduced slurry fluidity. Alomayri’s research [18] further corroborated that incorporating NA into geopolymer concrete (GPC) optimizes both mechanical properties and microstructural characteristics through matrix densification. Additionally, reducing the Si/Al ratio in geopolymers increases their specific surface area—a critical factor for adsorption-related applications. Alongside these nanomaterials, nano-calcium carbonate (NC) has garnered attention in cementitious material modification due to its cost-effectiveness and environmental compatibility. NC not only acts as a nano-filler improving matrix compactness [19], but its dissolution releases ${\mathrm{Ca}}^{2+}$ ions that participate in and accelerate the dissolution-reprecipitation processes of silicates and aluminosilicates. This promotes the formation of C-(A)-S-H gel or calcium-containing geopolymeric gel phases [20,21]. Studies confirm NC incorporation enhances early-age strength development and durability in both cementitious and select geopolymer systems [22]. Regarding nanomaterial interaction mechanisms, electrostatic behavior in alkaline environments warrants particular attention. Existing studies [23,24,25] indicate that in the highly alkaline environment of cement paste (pH > 12.5), silicate phases (e.g., hydration products of C_3_A/C_2_S) typically carry negative charges. In contrast, aluminate phases (e.g., hydration products of C_3_A) exhibit an isoelectric point near pH 9. Consequently, under cementitious high-pH conditions (pH ≈ 12.5), alumina (Al_2_O_3_) or aluminum-containing particles (e.g., calcined kaolin, calcium aluminate) tend toward positive or weakly charged states. The strong electrostatic attraction between these positively charged aluminum-containing particles/colloids (potentially derived from aluminum additives or their dissolution products) and negatively charged silicate particles is recognized as a key mechanism underlying rapid flocculation and even flash setting phenomena. Graphene oxide (GO) also demonstrates excellent modification effects. Studies have demonstrated [26,27,28,29] that trace amounts of GO (0.01–0.06% by weight) can significantly enhance the mechanical strength of concrete. GO is a layered nanomaterial composed of hydrophilic sheets rich in oxygen-containing functional groups (e.g., hydroxyl and epoxide groups on the basal planes, carbonyl and carboxyl groups at the edges), endowing it with an exceptionally high specific surface area and outstanding mechanical properties. Compared to carbon nanotube (CNT) reinforcement systems, GO achieves higher compressive strength gains at substantially lower dosages. This enhancement mechanism can be partially explained by the Water Film Thickness (WFT) theory [30,31,32]: Nanomaterials reduce the water film thickness on cement particle surfaces by competing for adsorption of free water. WFT is defined by the volume of free water (*V_m_*), the specific surface area of cement (*S_c_*), and the specific surface area of nanomaterials (*S_n_*). Crucially, the magnitude of WFT directly governs key material characteristics, including workability, mechanical performance, and durability.

(1) $$\delta =\frac{V_{m}}{S_{c}+S_{n}}$$

### 1.1. Research Significance

In summary, the specific surface area of nanomaterials plays a critical role in determining material performance. Current research predominantly focuses on the reinforcement effects of nanomaterials in fly ash/slag-based geopolymers (GP). However, coal gangue exhibits significantly lower reactivity due to its high content of inert crystalline quartz phases (>40%) and residual unburned carbon, which substantially diminishes the “catalytic-activation” efficiency of nanomaterials. A notable research gap persists regarding the mechanisms by which nanomaterials specifically activate inert SiO_2_/Al_2_O_3_ components in coal gangue, particularly due to the scarcity of in situ characterization evidence. To address this critical knowledge gap, this study aims to systematically investigate the influence patterns and underlying mechanisms of four distinct nanomaterials on the performance of coal gangue-based geopolymers (CGGP).

### 1.2. Research Objectives

This study selected four functionally distinct nanomaterials: nano-SiO_2_ (NS), graphene oxide (GO), nano-Al_2_O_3_ (NA), and nano-CaCO_3_ (NC). Through precise control of their incorporation levels (0.5–2.0 wt.%), we systematically evaluated their effects on the workability, setting time, and compressive strength of coal gangue-based geopolymers (CGGP). Advanced scanning electron microscopy coupled with energy-dispersive X-ray spectroscopy (SEM-EDS) was employed to elucidate the regulatory mechanisms of these nanomaterials on the geopolymerization process. This integrated approach aims to establish a clear structure-property relationship linking nanomaterial type, dosage, and performance. The findings are expected to provide both theoretical foundations and technical support for the high-value resource utilization of coal gangue and the development of green high-performance building materials.

## 2. Experimental Methods

### 2.1. Materials and Experimental Design

#### 2.1.1. Raw Materials

**Figure 1:**
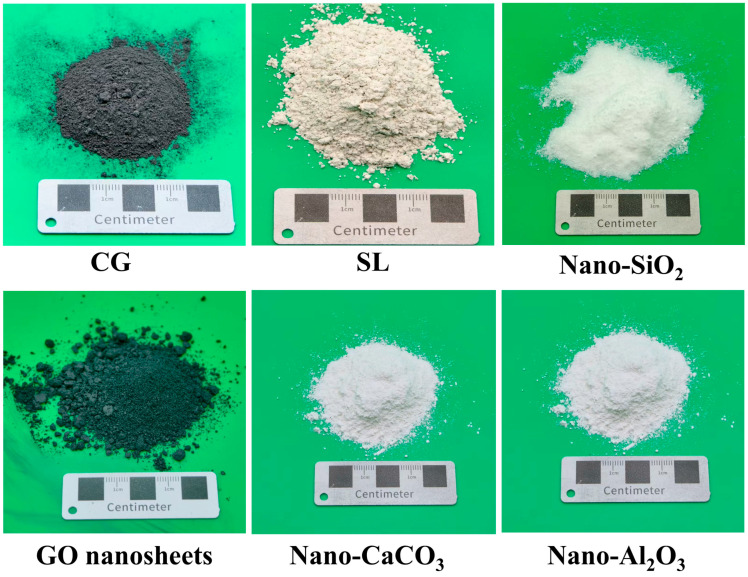
A variety of raw material appearances.

In this study, S95-grade slag powder (SL) was supplied by Dingnuo Purification Materials Co., Ltd. (Gongyi, China). The coal gangue (CG) was obtained from the Dayugou Coal Mine in Gongyi City, Zhengzhou, and subsequently ground in a ball mill for 30 min to achieve the desired fineness. Ordinary Portland cement (OPC, P.O 42.5) was provided by Conch Cement Co., Ltd. (Wuhu, China). The chemical compositions of these three raw materials are summarized in Table 1. NS was manufactured by Kete New Materials Technology Co., Ltd. (Shanghai, China). NA was supplied by the General Research Institute of Metals and Metallurgy (Beijing, China). GO was obtained from Metal Metallurgy Co., Ltd., (Guangzhou, China) and NC was produced by Boyu Gaoke New Material Co., Ltd. (Beijing, China). The physicochemical characteristics of the four types of nanomaterials used in the experiments are listed in Table 2. In addition, the morphological features of the nanomaterials, coal gangue powder, and slag powder are illustrated in Figure 1.

**Figure 2:**
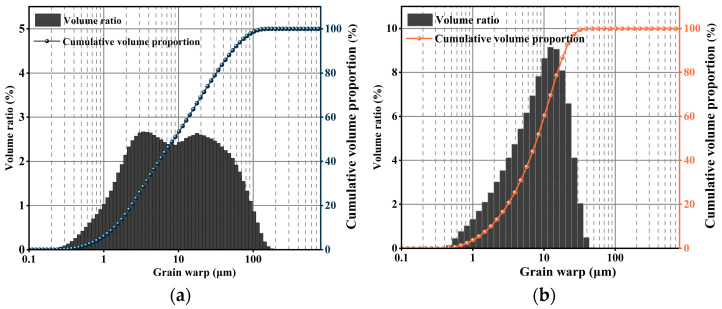
The particle size of (**a**) coal gangue and (**b**) slag.

**Figure 3:**
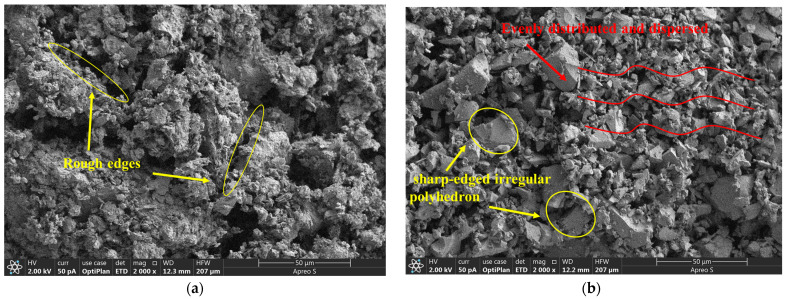
SEM micrographs of (**a**) coal gangue and (**b**) slag particles.

The particle size distributions of coal gangue powder and slag are presented in Figure 2. As shown in Figure 2a, the coal gangue exhibits a bimodal particle size distribution, with particle sizes mainly ranging from 1 to 100 μm. Two distinct peaks are observed at approximately 3 μm and 20 μm, corresponding to the enrichment of finer and coarser fractions, respectively. The relatively wide distribution is beneficial for close particle packing. In contrast, the results in Figure 2b indicate that the slag particles are predominantly distributed within the range of 1–30 μm, exhibiting a unimodal distribution. Compared with coal gangue, slag particles are generally smaller in size and display a more uniform and concentrated distribution. The microstructures of coal gangue powder and slag were further examined using scanning electron microscopy (SEM), the testing company is Shandong Qingdao Shendeng Chemical Technology Co., Ltd. (Qingdao, China). The specific model of the SEM instrument is Thermo Fisher Scientific’s ApreoS (Waltham, MA, USA), as shown in Figure 3. The coal gangue particles (Figure 3a) present irregular block-like morphologies with coarse edges, indicative of significant mechanical fragmentation during grinding. The surfaces are rough and characterized by numerous layered cracks and exfoliation structures, suggesting a multilayered mineral composition with partial interlayer separation. Localized particle agglomeration is also observed, where adhesion or stacking occurs between particles, possibly due to the presence of clay-like minerals or electrostatic interactions. The high surface roughness is expected to enhance interfacial reactivity between coal gangue and other components within the cementitious matrix. In contrast, slag particles (Figure 3b) exhibit fragmented, angular, and irregular polyhedral morphologies, with minimal particle agglomeration. The particles are relatively uniformly distributed and well-dispersed, which favors homogeneous mixing and reaction kinetics. Moreover, the overall low porosity of slag particles is advantageous for improving the compactness and strength development of the cementitious system.

#### 2.1.2. Mix Formulation and Sample Preparation Protocol

The alkaline activator was prepared by mixing sodium silicate solution (water glass), solid sodium hydroxide, and water. The mix proportions of the cementitious slurry are summarized in Table 3, comprising a total of 17 mixtures. Based on preliminary experiments, the optimal mix proportion for strength performance was determined as follows: The ratio of coal gangue, slag, and cement was fixed at 3:3:4. The modulus of the sodium silicate solution was adjusted to 1.7. The alkali-to-binder ratio, defined as the mass ratio of the alkaline activator to the coal gangue–slag–cement mixture, was maintained at 8%. The water-to-binder ratio, calculated as the mass of free water (including the free water in the sodium silicate solution) to the mass of the coal gangue–slag–cement mixture, was kept constant at 1.0. Four types of nanomaterials were incorporated, namely nano-SiO_2_ (NS group), graphene (GO group), nano-Al_2_O_3_ (NA group), and nano-CaCO_3_ (NC group). The dosages of the nanomaterials were varied in gradients from 0.5% to 2.0% by mass of the binder.

Fresh cementitious pastes were cast into standard cubic molds (40 mm × 40 mm × 40 mm) and compacted using an external vibrator to minimize entrapped air. After casting, the specimens were immediately covered with plastic film to prevent moisture loss [33,34,35]. All samples were demolded after 24 h and subsequently transferred to a standard curing chamber (20 ± 2 °C, relative humidity > 95%) until the designated testing ages of 3, 7, and 28 days.

### 2.2. Fluidity and Rheological Characterization

The fluidity of the fresh cement paste was measured using a mini-slump test, following the Chinese standard Test Method for Homogeneity of Concrete Admixtures (GB/T 8077-2023) [36]. The truncated cone mold had a height of 30 mm, a bottom diameter of 60 mm, and a top diameter of 36 mm. The mold was placed on a flat glass plate, filled with fresh paste, and then lifted vertically to allow the paste to spread freely into a disk shape within approximately 30 s. The diameters of the spread paste were measured along two perpendicular directions, and the average value was taken as the fluidity of the cement paste.

**Figure 4:**
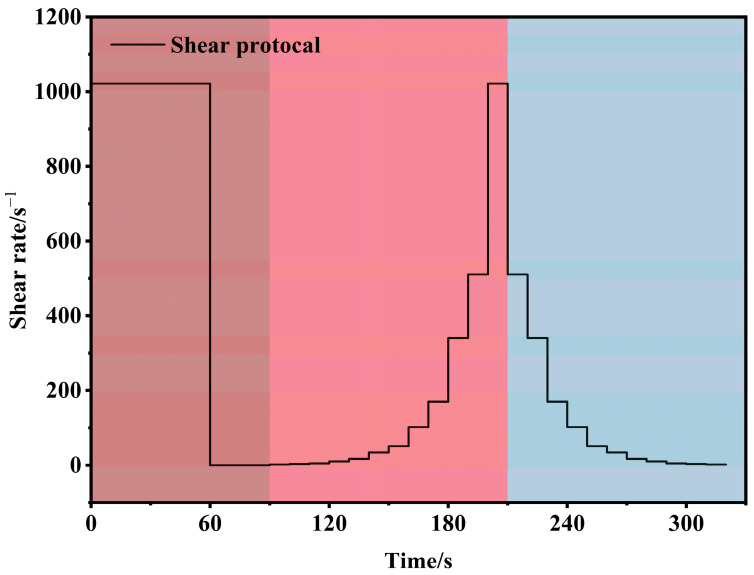
Shear procedure for rheological testing (brown, pre-shearing stage; red, shearing stage one; blue, shearing stage two).

The rheological properties of the composite slurry were tested using a digital rotational viscometer (MK-ZN12S, manufactured by Shandong Meike Instruments Co., Ltd., Qingdao, China). The test temperature was maintained at 25.0 ± 0.1 °C. To ensure a consistent initial state for all samples, the slurry was pre-sheared for 60 s at a constant shear rate of 1021.8 s^−1^ before formal testing. Rheological data were collected over two stages: (i) the upward curve was recorded from 90 s to 210 s, during which the shear rate was nonlinearly increased to a maximum of 1021.8 s^−1^; (ii) the downward curve was recorded from 210 s to 320 s, as the shear rate was nonlinearly decreased to 0 s^−1^. The entire rheological testing protocol is illustrated in Figure 4.

### 2.3. Setting Time and Uniaxial Compressive Strength Testing

Compressive strength tests were conducted in accordance with the Standard for Test Method of Basic Properties of Building Mortar (JGJ/T 70-2009) [37]. Cubic specimens with dimensions of 40 mm × 40 mm × 40 mm were tested at ages of 3, 7, and 28 days using a universal testing machine. For each mixture, three specimens were tested and the average value was reported as the compressive strength. The loading rate was controlled at 2.4 kN·s^−1^.

The setting times of standard-consistency cement paste were determined using a Vicat apparatus following Methods for Determination of Water Requirement for Normal Consistency, Setting Time and Soundness of Cement (GB/T 1346-2024) [38]. Fresh paste at standard consistency was placed into the Vicat mold set on a glass plate and immediately transferred to a humidity-controlled chamber. The time at which water was first added to the cement was recorded as the starting point. The first penetration measurement was performed based on the hardening progress; near the initial set, measurements were repeated at 5 min intervals (or shorter). The initial setting time was defined as the moment when the needle penetration stopped at 4 mm ± 1 mm above the base plate. Near the final set, measurements were taken at 15 min intervals (or shorter), ensuring that the needle did not penetrate into a previous hole. The final setting time was reached when the needle penetration was 0.5 mm, the annular attachment left no mark on the specimen surface, and the initial-setting needle penetrated no more than 1 mm into the small end face of the specimen. Both the initial and final setting times were confirmed by immediately repeating the measurement; only when the two consecutive results met the respective criteria were the times recorded.

### 2.4. Porosity and Water Absorption

The water absorption test was conducted in accordance with the American standard ASTM C642-13: Standard Test Method for Density, Absorption, and Voids in Hardened Concrete [39]. Mortar specimens with dimensions of 40 mm × 40 mm × 40 mm were used. After 28 days of curing, the specimens were oven-dried at 80 °C for 24 h and then weighed (*A*). Subsequently, the specimens were immersed in water for not less than 48 h, and their saturated surface-dry mass was recorded (*B*). The specimens were then boiled in a water bath for 5 h, cooled to room temperature, and weighed again (*C*). Finally, the apparent mass of each specimen suspended in water was measured (*D*). The calculation formulas are given as follows:

(2) $$Water\;adsorption=\;\frac{B\;-A}{A}\;\times 100\%,$$

(3) $$Porosity=\frac{C-A}{C-D}\times 100\%.$$

### 2.5. Microstructure Testing

The microstructural evolution and phase composition of the coal gangue-based geopolymers modified with four types of nanomaterials were characterized using scanning electron microscopy (SEM) combined with energy-dispersive spectroscopy (EDS). After mechanical strength testing, fractured specimens were randomly selected for microstructural analysis. For SEM observation (Thermo Fisher Scientific, Apreo S Waltham, MA, USA), a small amount of sample was affixed to a conductive adhesive and mounted on a sample holder, followed by evacuation to 5 × 10^−3^ Pa. After stabilizing for 2 min, imaging was performed. EDS analysis was conducted under an accelerating voltage of 10 kV to determine the elemental distributions in the regions containing dispersed nanomaterials.

**Figure 5:**
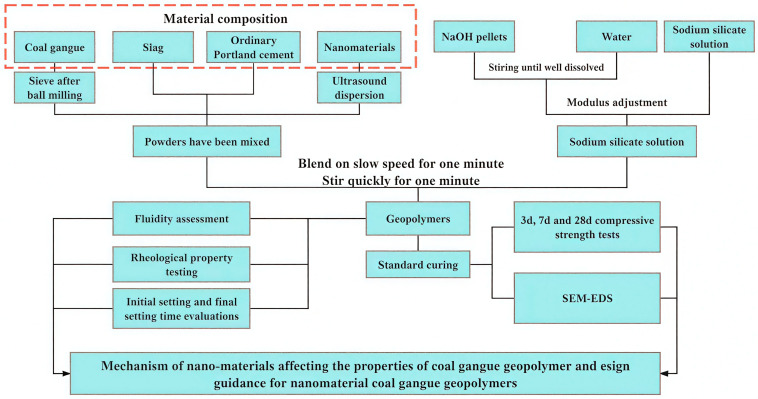
Workflow diagram.

To summarize the overall workflow—including material preparation, curing procedures, and characterization methods—a schematic flowchart is provided in Figure 5.

## 3. Results and Discussion

### 3.1. Fluidity and Rheological Behavior of Slurries with Different Nanomaterials

#### 3.1.1. Fluidity

**Figure 6:**
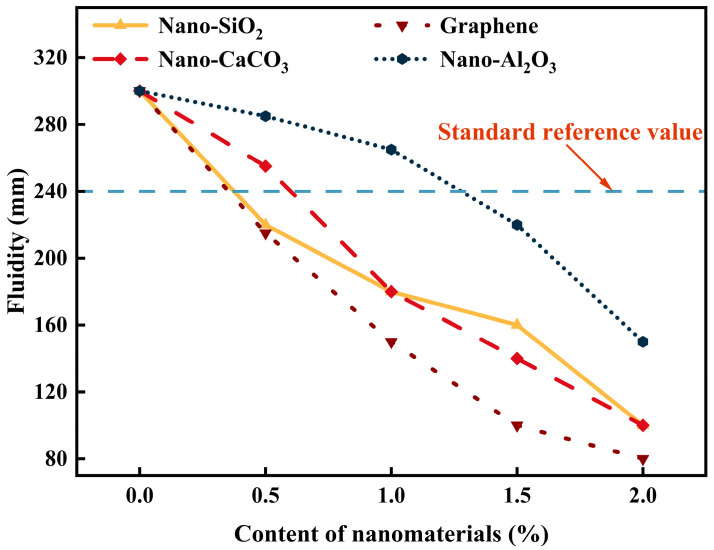
Fluidity of different nanomaterials at different dosages.

As shown in Figure 6, the incorporation of different nanomaterials had a pronounced influence on the fluidity of coal gangue-based geopolymers. Overall, the fluidity of the slurries decreased progressively with increasing nanomaterial dosage, although the extent of this reduction varied significantly depending on the type of nanomaterial. The addition of nano-SiO_2_ led to a continuous decrease in fluidity. When the dosage reached 1.0 wt.%, the fluidity dropped below 240 mm, which was lower than the theoretical reference value. This reduction can be mainly attributed to the strong hydrophilicity and large specific surface area of nano-SiO_2_, which adsorbs a considerable amount of free water on its surface, thereby increasing the viscosity of the slurry and significantly reducing its workability. Graphene oxide exhibited the most adverse effect on fluidity. With increasing dosage, the fluidity decreased sharply; at 2.0 wt.%, the fluidity was only 80 mm, representing a 73.33% reduction compared with the reference group (300 mm). This pronounced effect is ascribed to the extremely high specific surface area and layered structure of graphene oxide, which forms a three-dimensional network within the slurry. While such a structure improves the stability of the system, it also markedly enhances the internal cohesion, severely restricting flowability. The influence of nano-Al_2_O_3_ on fluidity was relatively mild at low dosages. However, when the dosage exceeded 1.0 wt.%, the reduction in fluidity became more pronounced; at 2.0 wt.%, the fluidity value was comparable to that of the same dosage of nano-SiO_2_. This indicates that nano-Al_2_O_3_ exerts little interference with slurry fluidity at low dosages, but its effect becomes considerably stronger at higher dosages. In contrast, nano-CaCO_3_ had the least effect on reducing fluidity across all tested dosages. At 0.5 wt.%, the fluidity was 285 mm, only 5% lower than that of the reference group (300 mm). This phenomenon can be mainly attributed to the relatively low surface free energy of nano-CaCO_3_ [40,41]. Compared with highly reactive nanomaterials such as nano-SiO_2_ and graphene oxide, nano-CaCO_3_ exhibits weaker competitive adsorption with water molecules in the alkaline geopolymer slurry, resulting in minimal free water consumption and only a slight increase in slurry viscosity.

#### 3.1.2. Rheological Behavior

Cement-based materials typically exhibit nonlinear rheological behavior under shear, primarily manifested as shear-thinning and shear-thickening phenomena. The mechanisms of shear-thinning are generally attributed to two main processes [42,43]. First, particle orientation: as the shear rate increases, particles within the suspension tend to preferentially align along the flow direction under hydrodynamic forces. Such orientation effectively reduces interparticle interactions (e.g., frictional resistance), thereby lowering the apparent viscosity of the slurry. Second, flocculation breakdown: the spatial flocculated network structures present in cement paste markedly hinder flow. However, when the applied shear stress (or shear rate) exceeds a critical threshold, these flocculated structures disintegrate into smaller floc units. During this process, bound water previously encapsulated within the flocculated structures is released into the continuous phase of the slurry as free water, which subsequently participates in the flow and enhances fluidity, thus reducing viscosity.

In contrast, shear-thickening typically occurs in suspensions with high solid volume fractions. Currently, the hydrocluster formation theory and particle inertia theory are widely applied to explain the increase in apparent viscosity with increasing shear rate [44].

The effects of four different types of nanomaterials on the rheological behavior of geopolymer pastes (water-to-binder ratio = 1.0) were investigated, in conjunction with previously established empirical rheological models [45,46,47,48]. The rheological responses at varying dosages of nanomaterials were fitted using both the Bingham model and the Herschel–Bulkley model. Depending on the shear regime, the flow behavior may exhibit either quasi-linear characteristics (Bingham-type) or nonlinear power-law characteristics (Herschel–Bulkley-type). The Bingham model assumes a constant viscosity beyond the yield stress and thus cannot capture the viscosity reduction (or increase) occurring in the high-shear region. In contrast, the Herschel–Bulkley model provides a better description of nonlinear flow behavior, but its fitting performance in the low-shear region is often less stable than that of the Bingham model. By employing both models, the yield stress can be cross-validated, and variations in plastic viscosity and flow index can be compared, thereby minimizing parameter distortion that may arise from relying on a single model.

The fitted rheological parameters for different nanomaterials are summarized in Table 4. For the nano-SiO_2_, CaCO_3_, and Al_2_O_3_ systems, both the Bingham and H-B models generally demonstrate good fitting performance (R^2^ > 0.95), indicating that the rheological behavior of these systems can, to some extent, be simplified as an ideal Bingham plastic fluid. However, when significant nonlinear structures develop within the system, the H-B model proves more advantageous due to its incorporation of the flow index parameter. For instance, at a 2 wt.% nano-SiO_2_ content, the R^2^ of the H-B model (0.951) is significantly higher than that of the Bingham model (0.785), demonstrating a deviation from linear rheological behavior and a better fit with the H-B model.

**Figure 7:**
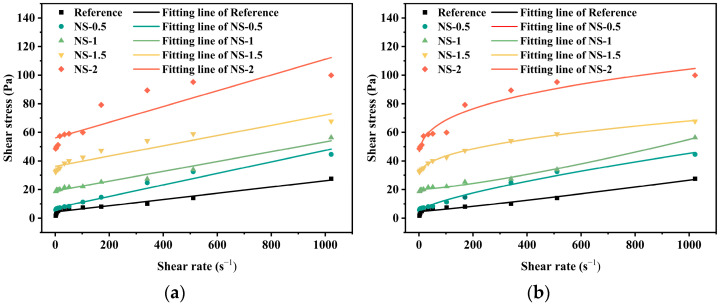
Rheological curves of different dosages of nano-silica and with different fitting models: (**a**) Bingham Model and (**b**) Herschel-Bulkley Model.

In contrast, the nano-graphene systems at all concentrations are more accurately described by the H-B model. Particularly at 1.5 wt.% graphene, the R^2^ of the Bingham model drops to 0.753, indicating strong nonlinear characteristics that render the Bingham model inapplicable. Therefore, the H-B model was established as the unified standard for cross-system comparison and mechanistic analysis in this study. The rheological behavior of coal gangue-based geopolymers was significantly influenced by both the type and dosage of the four nanomaterials, all of which increased the yield stress. The rheological curves of the geopolymer pastes with varying dosages are presented in Figure 7. As the dosage of nano-SiO_2_ increased from 0.5 wt.% to 2.0 wt.%, the Bingham yield stress rose markedly from 4.287 Pa to 36.255 Pa, corresponding to a 745% increase. This behavior can be attributed to the presence of surface silanol groups (Si–OH), which form covalent bonds with the geopolymer ${\left[{\mathrm{SiO}}_{4}\right]}^{4-}/{\left[{\mathrm{AlO}}_{4}\right]}^{5-}$ network, thereby reinforcing the gel skeleton (interfacial bonding theory [49]) and enhancing interparticle friction, leading to a higher shear stress. The plastic viscosity remained relatively stable (0.021–0.040 Pa·s), which is likely due to the spherical morphology of nano-SiO_2_ maintaining the consistency of plastic viscosity. At a dosage of 2.0 wt.%, the Herschel–Bulkley model exhibited a high fitting accuracy (R^2^ = 0.951), whereas the Bingham model showed a poorer fit (R^2^ = 0.785), suggesting that the slurry behavior at this dosage is closer to nonlinear rheological characteristics that cannot be adequately described by the Bingham model. The excellent fit of the H-B model (all R^2^ ≥ 0.951) confirms its applicability across all dosages. Notably, a flow index greater than 1 (*n* = 1.307) was observed only at the 1 wt.% addition level, indicating that the rheological behavior at this specific dosage was predominantly governed by shear thickening. A reasonable explanation for this phenomenon requires a comprehensive consideration of the interfacial chemistry of the nanoparticles and hydrodynamic effects: The strong covalent bonds formed between the Si–OH groups on the nanoparticle surfaces and the geopolymer network are the key factor leading to the significant increase in yield stress (τ_0_). However, at the critical dosage of 1 wt.%, the number of particles is sufficient to trigger a unique hydrodynamic response under high shear rates. At this point, particle motion becomes intense, and the physical interactions (e.g., hydrogen bonding) on their surfaces are no longer adequate to maintain a stable weak network. Instead, as particles move too rapidly, they collide and undergo mechanical interlocking, forming temporary rigid structures that result in shear thickening.

In other words, in the low-shear region, interfacial chemistry dominates and provides the high τ_0_, whereas in the high-shear region, the physical packing and frictional effects of particles dominate, leading to the shear-thickening behavior (*n* > 1). In contrast, at non-critical dosage levels, reversible weak structures (such as hydrogen bonds) play a dominant role in the rheological behavior, and their disruption leads to pronounced shear thinning. On the other hand, nanoparticles align along the flow direction within the shear field, thereby reducing flow resistance.

**Figure 8:**
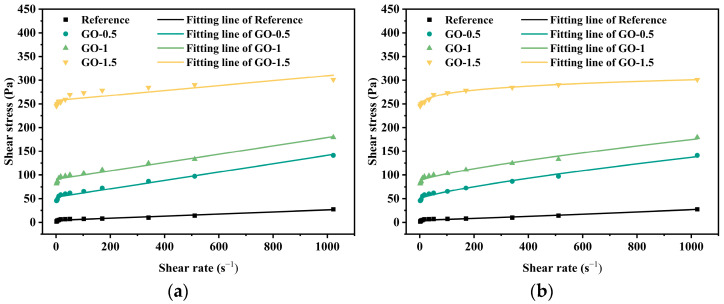
Rheological curves of different dosages of nanographene and with different fitting models: (**a**) Bingham Model and (**b**) Herschel-Bulkley Model.

The rheological curves of geopolymer pastes with varying graphene dosages are presented in Figure 8. It is noteworthy that for the GO-2 group (the sample with the highest graphene oxide dosage), the shear stress rapidly reached and triggered the upper limit of the rheometer (613.2 Pa) during testing, resulting in torque overflow. Due to the inability to obtain a complete stress distribution profile at high shear rates, this specific group could not be subjected to reliable fitting analysis using rheological models (e.g., Bingham or Herschel–Bulkley models). This outcome is highly consistent with the flow table test results, reflecting a significant enhancement in the internal structural strength of the paste by the high GO dosage. This is primarily attributed to the large specific surface area and strong surface adsorption of the GO nanosheets, which facilitate the formation of a dense network structure within the matrix. Such a network leads to a sharp increase in yield stress and flow resistance, ultimately surpassing the detection range of conventional instrumentation. According to the Herschel–Bulkley (H–B) model fitting, the yield stress of the reference group (without nanomaterials) was 4.567 Pa. When graphene was incorporated at 0.5 wt.%, the yield stress increased dramatically to 49.527 Pa, representing a 984% increase. Among the four nanomaterials examined, graphene exhibited the strongest influence on rheological behavior. At dosages of 0.5, 1.0, and 1.5 wt.%, the yield stresses were 49.527 Pa, 87.415 Pa, and 222.295 Pa, respectively. The yield stress exhibited an almost exponential increase with dosage. This trend can be explained by the sheet-like structure of graphene: at low concentrations, graphene nanosheets are well-dispersed and largely isolated, with minimal contact, allowing easier relative sliding under stress, thus resulting in lower yield stress. As the graphene content increased, the interlayer spacing decreased, leading to sheet-to-sheet overlap. At these contact points, van der Waals forces, π–π stacking, or mechanical interlocking create junctions. Once a sufficient number of junctions form and extend throughout the system, a continuous three-dimensional graphene network develops. This network shifts the primary load-bearing mechanism from the geopolymer matrix to the graphene skeleton, thereby sharply increasing yield stress. At 1.5 wt.% graphene, the Bingham model fitting accuracy was poor (R^2^ = 0.753), whereas the H–B model provided a more suitable description, confirming that graphene-modified pastes consistently exhibited shear-thinning behavior. Under static or low-shear conditions, graphene nanosheets are randomly dispersed in the matrix, forming spatial networks stabilized by van der Waals forces and hydrogen bonding. These networks impede flow, significantly increasing the initial viscosity. At higher shear rates, however, the applied shear forces reorient graphene nanosheets along the flow direction [42,43], disrupting the network structure, lowering viscosity, and reducing flow resistance. Additionally, during geopolymer gelation, Si–O–Al networks (≡Si–O–Al≡) and unreacted particles form weakly bonded flocculated structures or agglomerates. At low shear, these structures increase viscosity, whereas at high shear, they are broken apart, releasing entrapped water and thereby further reducing flow resistance.

**Figure 9:**
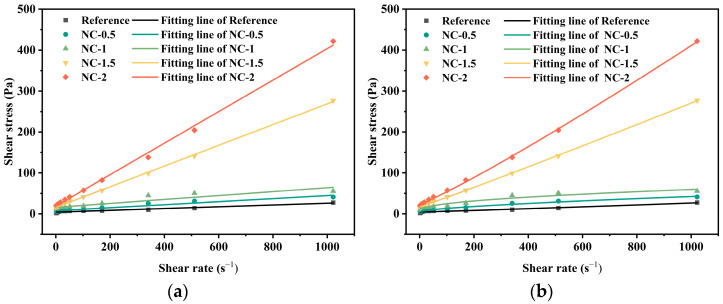
Rheological curves of different dosages of nano-calcium carbonate with different fitting models: (**a**) Bingham Model and (**b**) Herschel-Bulkley Model.

**Figure 10:**
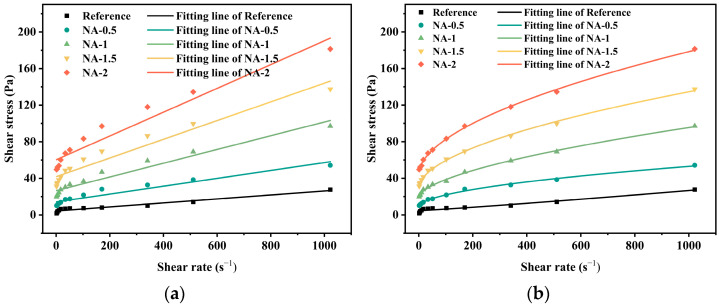
Rheological curves of different dosages of nano-alumina with different fitting models: (**a**) Bingham Model and (**b**) Herschel-Bulkley Model.

The rheological curves of geopolymer pastes with different dosages of nano-CaCO_3_ are shown in Figure 9. Overall, both the Bingham and Herschel–Bulkley models provided good fits for the nano-CaCO_3_ and nano-Al_2_O_3_ systems. For the nano-CaCO_3_ system, the yield stress exhibited a gradual increase with increasing dosage. Although ${\mathrm{Ca}}^{2+}$ released from CaCO_3_ promotes coagulation [23,24,25], the spherical morphology of nano-CaCO_3_ reduces internal friction, thereby offsetting part of the shear stress increment induced by Ca^2+^ coagulation. As the dosage increased from 0.5 wt.% to 2.0 wt.%, the yield stress increased from 7.639 Pa to 18.777 Pa, corresponding to a 146% growth. This indicates that nano-CaCO_3_, acting as rigid spheres, enhances the geopolymer skeleton through physical filling, but its reinforcing efficiency is lower than that of nano-SiO_2_ (745% growth). Except at 2 wt.% dosage, where the flow index reached *n* = 1.107, all other groups exhibited *n* < 1, confirming shear-thinning behavior. The rheological curves of the nano-Al_2_O_3_ system are presented in Figure 10. The yield stress obtained from the Bingham model increased significantly from 14.122 Pa (0.5 wt.%) to 60.225 Pa (2 wt.%), representing a 326% increase, which is markedly higher than that of the CaCO_3_ system (146%). According to the water film thickness theory 303132, the higher specific surface area of nano-Al_2_O_3_ strengthens interfacial bonding, while its higher surface energy and reactivity further promote cross-linking of the geopolymer network. At all dosages, the Al_2_O_3_-modified pastes exhibited shear-thinning behavior, as the nanoparticle–geopolymer networks disintegrated under shear. The relatively low flow index values (*n* = 0.523–0.597) indicate that the Al_2_O_3_ networks are more susceptible to shear-induced breakdown, suggesting a stronger thixotropic character compared with the CaCO_3_ system.

### 3.2. Compressive Strength

**Figure 11:**
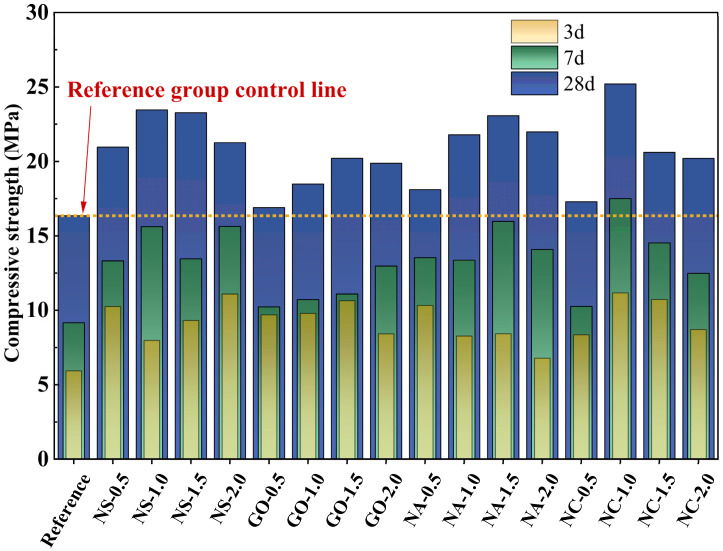
Uniaxial compressive strength for each group.

The uniaxial compressive strengths of different groups at various curing ages are presented in Figure 11. The reference group (without nanomaterial incorporation) exhibited relatively low compressive strengths at 3, 7, and 28 days, indicating slower strength development and lower ultimate strength. The NC-1.0 group exhibited the highest 28-day compressive strength, showing an increase of 54.18% compared to the reference group. In contrast, NS-1.0, NS-1.5, and NS-2.0 all showed significantly higher compressive strengths, particularly at 28 days, where the specimens with higher dosages of nano-SiO_2_ (1.5 and 2.0 wt.%) exhibited more pronounced improvements. At both 3 and 7 days, the compressive strength was also much higher than that of the reference group, suggesting that nano-SiO_2_, through its filling effect and the promotion of hydration products such as C–S–H gel (and calcium hydroxide), substantially enhanced both early-age and long-term strengths [50,51,52]. For the graphene-modified groups (GO-1.0, GO-1.5, and GO-2.0), compressive strengths were slightly higher than those of the reference group, especially at 3 and 7 days, but the overall improvement was inferior to that of the nano-SiO_2_ groups. The enhancement is attributed to graphene’s excellent thermal conductivity and crack-bridging capacity, which helps mitigate microcrack propagation. However, due to its poor dispersibility, graphene tends to agglomerate at higher dosages, limiting its strengthening efficiency. The nano-Al_2_O_3_ groups (NA-1.0 and NA-1.5) exhibited moderate improvements, particularly at 28 days, whereas the high-dosage group (NA-2.0) showed a decline in compressive strength. This behavior may be related to the reactivity and filling capacity of nano-Al_2_O_3_, where excessive addition could lead to the formation of undesirable reaction byproducts or disruption of the internal microstructure, thus offsetting its reinforcing effect. For the nano-CaCO_3_ groups (NC-1.0 and NC-1.5), a slight strengthening effect was observed, but the overall improvement was less significant. At 2.0 wt.% dosage, the compressive strength was nearly identical to that of the reference group. This can be explained by the fact that nano-CaCO_3_ mainly serves as a filler to improve paste compactness, while its relatively low reactivity limits its influence on the geopolymerization process. At excessively high dosages, over-densification may hinder hydration reactions, thereby reducing the overall strength development. Overall, the optimal dosage range for most nanomaterials was between 1.0 and 1.5 wt.%. Excessive addition led to particle agglomeration and reduced dispersion, which in turn impeded the strengthening effect rather than enhancing it.

### 3.3. Setting Time

**Figure 12:**
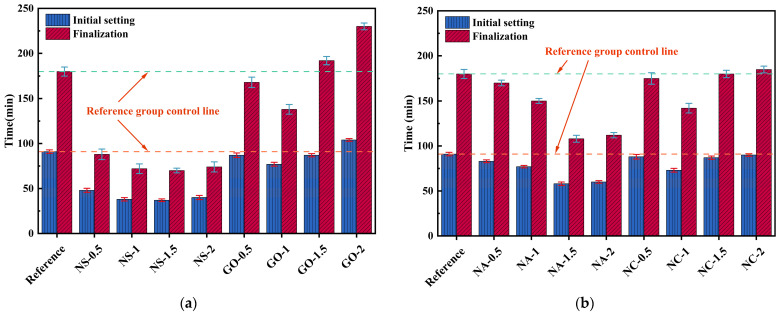
The initial and final coagulation times for the experimental groups: (**a**) Nano silica and nanographene; and (**b**) Nano alumina and nano calcium carbonate.

Figure 12 illustrates the effects of different types of nanomaterials (SiO_2_, graphene, Al_2_O_3_, and CaCO_3_) at dosages ranging from 0.5 to 2.0 wt.% on the initial and final setting times of the cementitious system. The results indicate strong dependence on both material type and dosage, with distinct regular trends. Nano-SiO_2_ exhibited a pronounced accelerating effect on setting. At 1.5 wt.%, the initial setting time decreased from 91 min (reference group) to 37 min, corresponding to a 59.3% reduction, while the final setting time shortened from 180 min to 70 min, a 61.1% reduction. At 2.0 wt.%, the initial setting time slightly rebounded to 40 min, and the final setting time increased to 74 min, though both remained ~55% shorter than those of the reference group. This demonstrates that nano-SiO_2_ consistently promoted setting across the 0.5–2.0 wt.% range, with the most effective range being 1.0–1.5 wt.%. The strong accelerating effect can be attributed to the extremely high specific surface area and reactivity of nano-SiO_2_, which rapidly reacts with ${\mathrm{Ca}}^{2+}$ to form C–S–H gel and provides abundant nucleation sites. These factors accelerate the transition from a liquid suspension to a structural gel. However, at higher dosages, particle agglomeration may occur, weakening the accelerating effect. Graphene showed a “first acceleration, then retardation” trend. At 1.0 wt.%, the initial setting time decreased to 61 min, a reduction of 32.9% compared with the reference, while the final setting time shortened to 138 min, a 23.3% reduction. In contrast, at 2.0 wt.%, the initial setting time increased to 104 min (+14.3%), and the final setting time was prolonged to 230 min (+27.8%), indicating a significant retardation effect. At low dosages, graphene’s excellent thermal conductivity and layered structure may enhance local heat accumulation and spatial structuring, thereby slightly accelerating hydration. However, at higher dosages, graphene tends to agglomerate and encapsulate cementitious particles, hindering ion migration and water penetration, thus inhibiting hydration reactions and delaying the setting process.

For the nano-Al_2_O_3_ system, the strongest accelerating effect was observed at 1.5 wt.%, where the initial setting time decreased to 58 min (a 36.3% reduction compared with the reference), and the final setting time shortened to 108 min (a 40.0% reduction). Although the accelerating effect of nano-Al_2_O_3_ was slightly weaker than that of nano-SiO_2_, its performance was more stable. This can be attributed to the high surface energy and nucleation ability of Al_2_O_3_ particles, which promote the early formation of aluminate ferrite tri-sulfate (AFt) and other aluminate hydrates, as well as ion precipitation reactions. However, due to its lower reactivity compared with SiO_2_, the reaction kinetics of Al_2_O_3_ are more strongly governed by the alkalinity of the system. Nano-CaCO_3_ exhibited only a weak accelerating effect. At 1.0 wt.%, the initial setting time decreased to 73 min (a 19.8% reduction), and the final setting time shortened to 142 min (a 21.1% reduction). However, at 2.0 wt.%, the accelerating effect was almost negligible, with an initial setting time of 90 min and a final setting time of 185 min, both close to the reference values. This limited contribution is mainly because CaCO_3_ participates minimally in chemical reactions; instead, it primarily serves as a nucleation template that improves the spatial distribution of hydration products. Nevertheless, its lack of intrinsic reactivity results in only modest setting acceleration.

### 3.4. Porosity and Water Absorption

**Figure 13:**
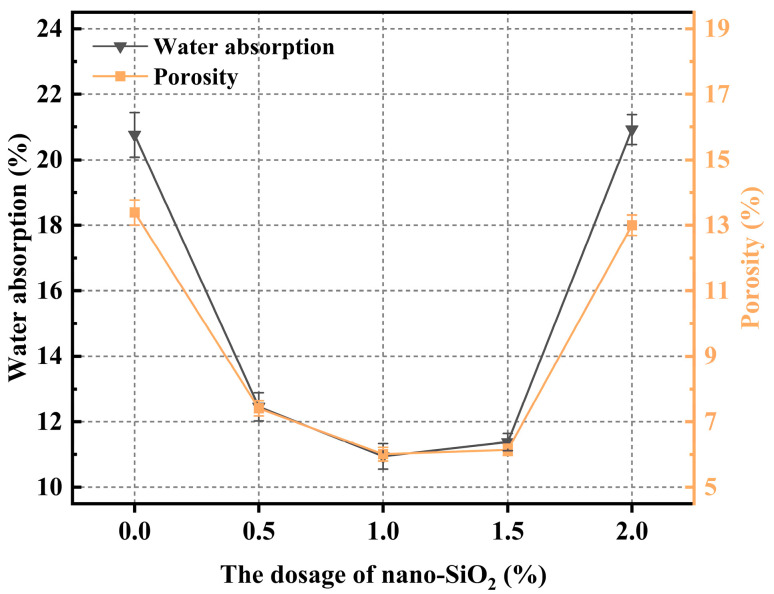
The porosity and moisture content vary with the amount of nano-SiO_2_ content.

Porosity represents the total volume of voids within the material, while water absorption reflects the ability of these pores to retain water. Under theoretical limiting conditions, open porosity and water absorption exhibit a linear relationship. Experimental results generally demonstrate a positive correlation, and several researchers [53,54] have proposed and validated typical porosity–water absorption models for geopolymers. The present study further confirms this positive correlation between porosity and water absorption in geopolymer systems. It should be noted that while the pore size distribution and specific pore types were not quantified in this study, the observed changes in total porosity primarily stem from the dual effect of physical gap-filling and the introduction of the filler’s intrinsic micro-porosity. Figure 13 shows the effects of nano-SiO_2_ dosage on the porosity and water absorption of coal gangue-based geopolymer (CGGP). Both porosity and water absorption initially decreased and then increased with increasing nano-SiO_2_ content. At a dosage of 1.0 wt.%, the water absorption and porosity reached their minimum values of 10.94% and 6.01%, respectively. Compared with the reference group (20.76% and 13.39%), these values decreased by 47.27% and 55.11%, indicating a significant densification of the microstructure induced by nano-SiO_2_ at the optimal dosage.

**Figure 14:**
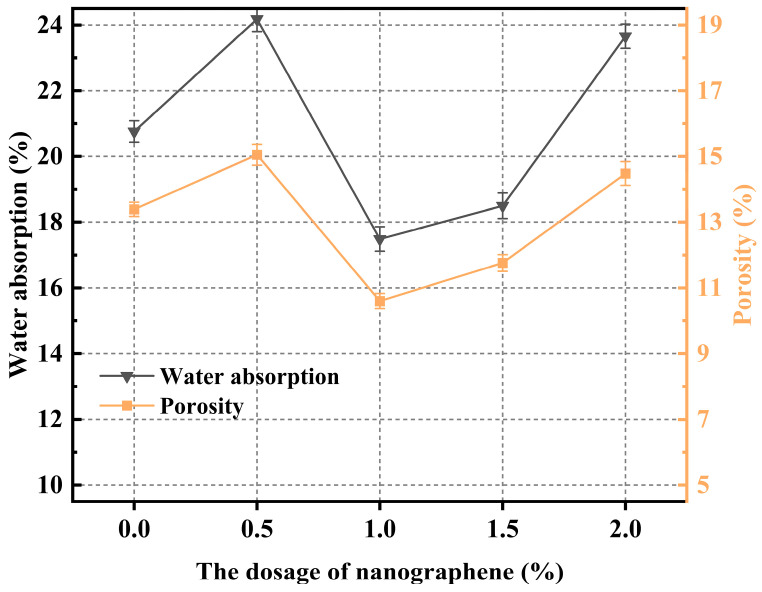
The porosity and moisture content vary with the amount of nanographene content.

Figure 14 depicts the variations in porosity and water absorption of CGGP with increasing graphene dosage. Both parameters decreased initially and then increased with further addition of graphene. At 1.0 wt.%, the lowest values were observed: water absorption of 17.49% and porosity of 10.60%, representing reductions of 15.77% and 20.83%, respectively, compared with the reference group. At 0.5 wt.%, however, water absorption and porosity increased to 24.18% and 15.05%, which were 16.47% and 12.40% higher than those of the reference. Similarly, at 2.0 wt.%, water absorption and porosity were 23.66% and 14.48%, corresponding to increases of 14.0% and 8.1%, respectively. These results suggest that both low (0.5 wt.%) and high (2.0 wt.%) graphene dosages increase porosity and water absorption relative to the blank group, whereas only appropriate dosages (1.0–1.5 wt.%) yield reductions below the reference values. The underlying mechanism is related to the dispersion state of graphene nanosheets. At low dosages (e.g., 0.5 wt.%), graphene tends to agglomerate due to poor dispersibility, forming clusters that act as “pore nuclei” or disturb slurry fluidity, resulting in structural defects and microcracks that elevate porosity and water absorption. At moderate dosages (1.0–1.5 wt.%), graphene can be more uniformly dispersed within the matrix, acting as a “nano-reinforcing skeleton” that facilitates the oriented nucleation of hydration products on graphene surfaces and promotes the formation of a denser gel structure. At high dosages, however, the dispersion of graphene deteriorates significantly, and severe agglomeration occurs. These agglomerates create localized stress concentrations or voids, introducing defects that ultimately increase porosity and water absorption.

**Figure 15:**
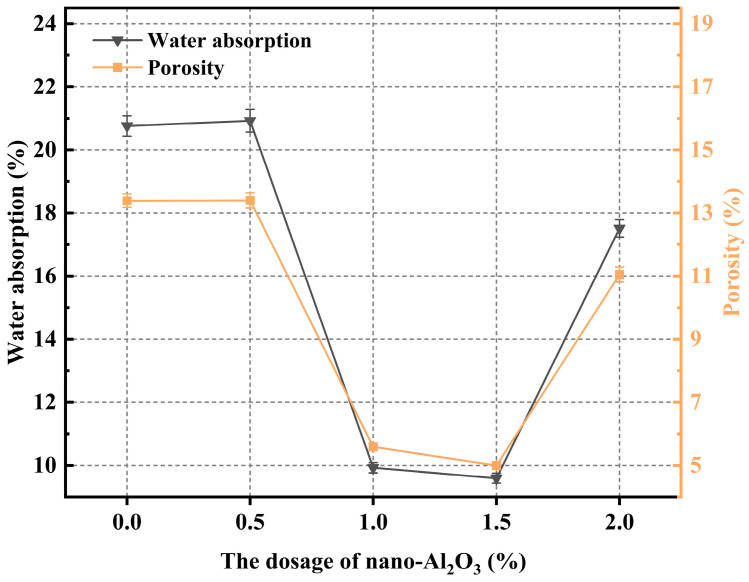
The porosity and moisture content vary with the amount of nano-Al_2_O_3_ content.

Figure 15 illustrates the variations in porosity and water absorption of CGGP with different dosages of nano-Al_2_O_3_. Both parameters decreased initially and then increased with increasing Al_2_O_3_ content. At 0.5 wt.%, the values were nearly identical to those of the reference group, indicating that the effect of nano-Al_2_O_3_ was negligible at this dosage. The minimum values were observed at 1.5 wt.%, with water absorption and porosity reduced to 9.49% and 5.00%, respectively. Compared with the reference group, these values decreased by 54.26% and 62.66%, representing the lowest porosity and water absorption among all tested groups. At the lower dosage of 0.5 wt.%, the poor dispersibility of nano-Al_2_O_3_ and the insufficient dosage may have prevented the nanoparticles from reaching the critical threshold required to fully exert their filling and “nano-reinforcing skeleton” effects. When the dosage was further increased beyond 1.5 wt.%, particle agglomeration became more pronounced due to deteriorated dispersibility, leading to less effective densification of the matrix and, consequently, higher porosity and water absorption.

**Figure 16:**
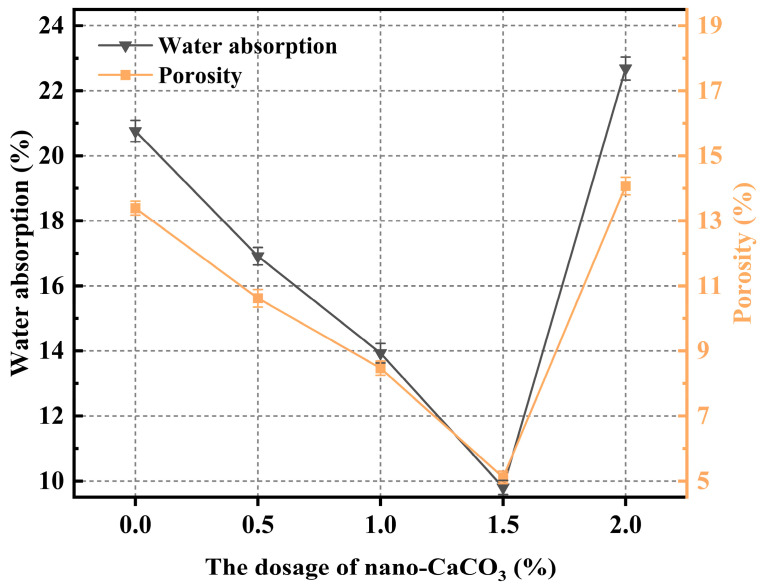
The porosity and moisture content vary with the amount of nano-CaCO_3_ content.

Figure 16 shows the variations in porosity and water absorption of CGGP with increasing nano-CaCO_3_ dosage. Both parameters first decreased and then increased as the CaCO_3_ content increased. The minimum values were obtained at 1.5 wt.%, where the water absorption and porosity reached 9.81% and 5.11%, respectively. Compared with the reference group, these values decreased by 52.76% and 61.83%. However, when the dosage was further increased to 2.0 wt.%, both porosity and water absorption exceeded those of the reference group, indicating that excessive addition of nano-CaCO_3_ may introduce defects or hinder hydration, thereby compromising the densification effect.

### 3.5. Microstructural Analysis

**Figure 17:**
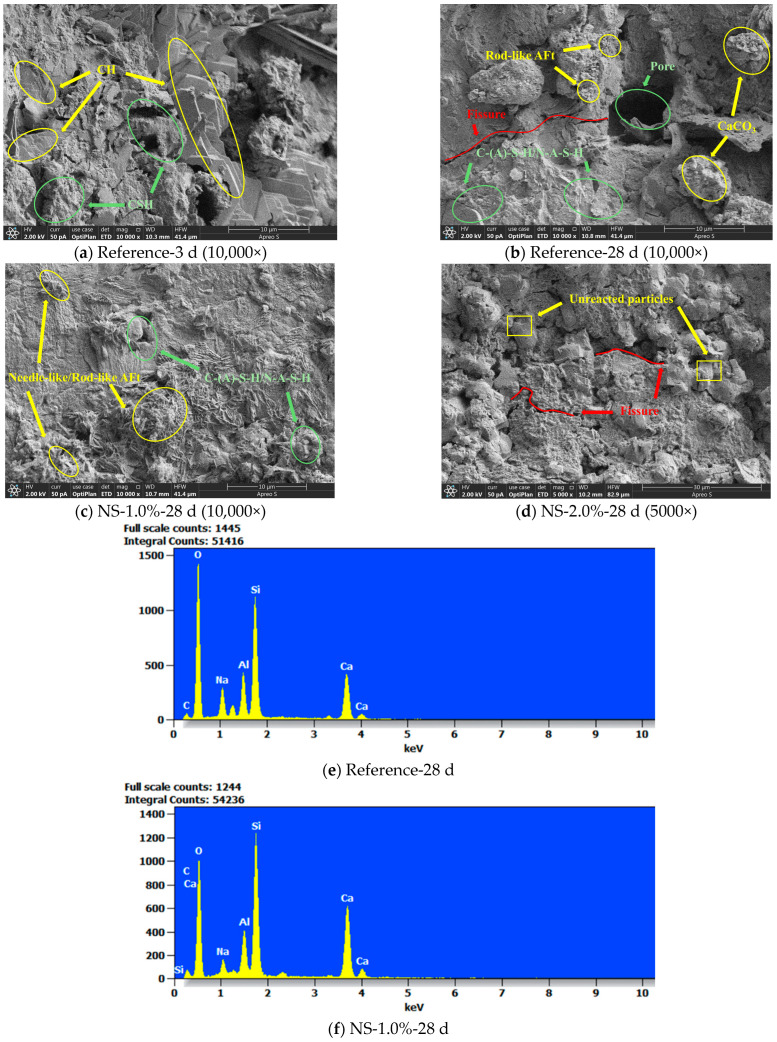

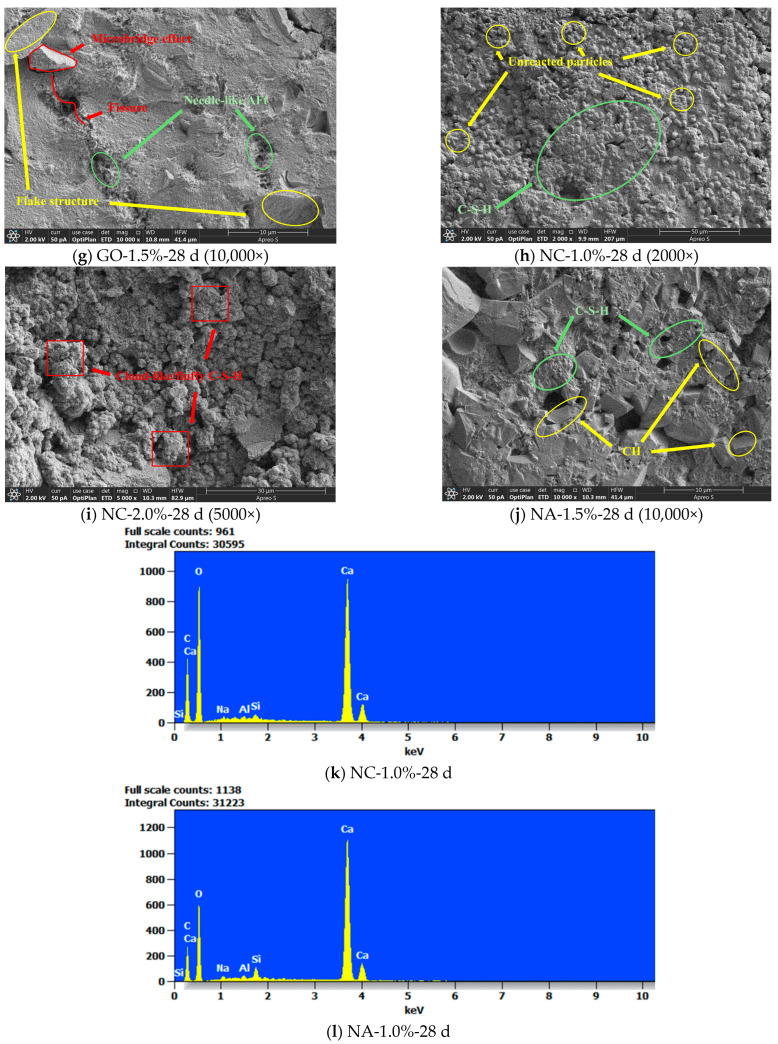
SEM and EDS results of each test group.

Figure 17a shows the SEM image of the Reference group after 3 days of curing. A typical feature is the abundant presence of CH (calcium hydroxide) crystals, which appear stacked in layers. The CH crystals exhibit regular morphology with well-defined edges, while C–S–H is mainly observed in flocculent or clustered forms. This can be explained by the fact that at the early hydration stage, the reaction is dominated by Portland cement clinker, in which the development of the C–S–H gel structure is relatively slow. Meanwhile, the hydration of C_3_S and C_2_S rapidly releases ${\mathrm{Ca}}^{2+}$ and ${\mathrm{OH}}^{-}$ ions, leading to the fast formation of CH. Furthermore, due to the absence of nanomaterial additives in the Reference group, the system is relatively loose and lacks sufficient nucleation “competition” sites, allowing CH crystals to freely grow into plate-like or hexagonal morphologies in open spaces. Figure 17b presents the SEM image of the Reference group at 28 days. Compared with the 3-day microstructure, porosity decreased but was still evident, and distinct microcracks remained, indicating that the structure was not yet fully densified, though substantially improved compared with the early age. Hydration products observed include small amounts of rod-like AFt, as well as C-(A)-S-H gel (calcium–aluminum–silicate–hydrate) and N-A-S-H gel (sodium–aluminum–silicate–hydrate). Because C-(A)-S-H and N-A-S-H often coexist, their morphologies are difficult to distinguish clearly. The formation of C-(A)-S-H results mainly from reactions between ${\mathrm{Ca}}^{2+}$, SiO_2_ and Al_2_O_3_ in slag and coal gangue with the alkaline activator, and it serves as the dominant binding phase in alkali-activated systems, contributing significantly to later-age strength. Meanwhile, the high silica–alumina content in coal gangue reacts with ${\mathrm{Na}}^{+}$ to generate N-A-S-H gel, which provides auxiliary binding capacity. In addition, CH partially carbonated into CaCO_3_. AFt was not a dominant product in this alkali-activated system; only small quantities were detected. Its presence originates from the gypsum in cement, which provides the sources ${\mathrm{Ca}}^{2+}$, ${\mathrm{Al}}^{3+}$, ${\mathrm{SO}}_{4}^{2-}$. Over time, additional ${\mathrm{Al}}^{3+}$ released from coal gangue and cement contributed to the delayed formation of AFt at 28 days, since the highly alkaline environment suppressed its early generation (delayed reaction effect).

Figure 17c shows the SEM image of the NS-1.0 group after 28 days of curing. The microstructure appears dense with very few pores; crystals are abundant and well-developed, with orderly alignment. A large number of rod- and needle-like crystals are clearly observed, covered by a dense gel matrix. Different hydration products are distributed relatively uniformly, and the bonding between particles is compact. Notably, at a dosage of 1.0 wt. %, nano-SiO_2_ produced the highest compressive strength and the lowest porosity within the NS series. This can be attributed to the fact that nano-SiO_2_ actively participates in the pozzolanic reaction, accelerating the formation rate and increasing the quantity of C-(A)-S-H gel, while also improving the Si/Ca ratio of the binder gel system, thus contributing to the development of a more stable structure.

Nano-SiO_2_ particles penetrate into micro-pores and fill voids, effectively reducing capillary pores and improving structural compactness. Moreover, nano-SiO_2_ promotes the generation of AFt: on the one hand, its extremely high specific surface area and surface reactivity enhance early-stage hydration of cement, slag, and coal gangue, accelerating mineral dissolution and cement hydration, thereby releasing additional ${\mathrm{Ca}}^{2+}$ (as required for AFt framework formation). On the other hand, nano-SiO_2_ serves as a nucleation site, providing stable positions for AFt crystallization. As a result, AFt forms more complete needle- and rod-like morphologies, which are more easily observed in SEM images. The crystals are uniformly aligned and well-ordered, indicating that the crystallization process is optimized under the nucleation induction effect of nano-SiO_2_ particles. Figure 17d shows the SEM morphology of the sample with 2% nano-SiO_2_ content after 28 days of curing. This image provides direct micro-evidence for explaining the significant reduction in macroscopic mechanical strength observed in this group of samples. The fundamental reason for the strength degradation can be unequivocally attributed to the serious agglomeration of NS observed in the figure. As shown in the image, large blocky unreacted particles remain on the specimen surface, accompanied by irregular pores and crack-like structures, while the hydration products in certain regions are sparsely distributed. The overall structure appears porous and coarse, displaying a characteristic of a “loose skeletal framework.” Although in theory a higher dosage of nano-SiO_2_ should provide more nucleation centers and reactive silica species, at 2 wt.%, the nanoparticles underwent severe agglomeration, which significantly reduced their dispersibility and reactivity. Consequently, the effective amount of SiO_2_ actually participating in the reaction decreased. In addition, some SiO_2_ particles were found to coat the surfaces of other raw materials, forming “shell-like structures,” which further inhibited the dissolution of reactive species from slag and coal gangue. This phenomenon led to a reduction in the formation capacity of binding phases (such as C-(A)-S-H and N-A-S-H), preventing the effective filling of pores and microcracks, thereby increasing porosity and weakening structural compactness. The SEM images also reveal numerous coarse, plate-like particles with clear edges, which are presumed to be unreacted or insufficiently activated slag and coal gangue residues. At this dosage, nano-SiO_2_ aggregates blocked the pathways between reactive particles or clustered together, restricting the diffusion of hydration products and leaving many particles in an “inactive” state. As a result, internal voids expanded, microcracks proliferated, and the transformation into effective hydration products was hindered, leading to the deterioration of microstructural densification.

Figure 17e shows the EDS spectrum of the Reference group after 28 days of curing. The major elements detected were Ca, Si, Al, and O. The Ca/Si ratio was relatively low, while the Si peak intensity was comparatively high, indicating that a large amount of silicate hydrates had formed. The Al peak was of medium intensity, mainly originating from the alumina in coal gangue or slag, and contributed to the formation of aluminosilicate hydrates. The presence of Al clearly confirms the formation of C-(A)-S-H gel as the main hydration product of alkali-activated reactions, which is also the primary source of strength development in the system. Figure 17f presents the EDS spectrum of the NS-1.0 group at 28 days. The Si peak intensity was significantly higher than that of the Reference group, reflecting the contribution of nano-SiO_2_ addition. The increased reactive Si content originated both from the external incorporation of nano-SiO_2_ and from the enhanced dissolution of silicate species in slag and coal gangue induced by the presence of SiO_2_. This increase in available Si markedly promoted the formation of C-(A)-S-H and N-A-S-H gels, which played a crucial role in improving the compactness and strength of the system. The Ca peak intensity was moderate, indicating widespread participation of Ca in the hydration reactions. In the alkali-activated matrix, Ca mainly originated from cement and slag. With the presence of nano-SiO_2_, Ca combined with external Si to form C–S–H and C-(A)-S-H gels, while also contributing to the precipitation of a certain amount of AFt crystals. The elevated Ca peak intensity further suggests that part of the Ca participated in the formation of more stable crystalline hydration products, thereby enhancing the structural stability of the system. While EDS provides insights into the elemental distribution of the matrix, its results should be interpreted with caution. In this study, the identified phases are characterized based on a synthesis of morphology, elemental ratios, and established literature, acknowledging that EDS alone does not constitute definitive phase validation.

Figure 17g presents the micro-morphology of the sample incorporating 1.5 wt.% nano-graphene oxide (GO) (GO-1.5% group) after 28 days of curing. The SEM image reveals a dense overall structure with no significant interconnected pores or extensive microcracks. Furthermore, a substantial amount of needle-like ettringite (AFt) crystals can be observed interwoven within the matrix, collectively forming a continuous and well-integrated microstructure. The incorporation of GO, characterized by its high specific surface area and surface energy (measured to be approximately 65–80 mJ/m^2^), significantly promotes its dispersion and interfacial interactions within the cement paste. This high surface energy enables the 1.5 wt.% GO to effectively form a three-dimensional conductive network skeleton within the hydration products and exert a “micro-bridging” effect, directly enhancing the interconnectivity between C-S-H gel particles. Figure 17h shows the SEM image of the NC-1% group after 28 days of curing. A large area of continuous and finely textured C-S-H gel is visible, indicating ongoing growth and pore-filling by the C-S-H gel. Within the image, locally distinct crystalline structures, identified as calcium hydroxide (CH) crystals and ettringite (AFt) among other hydration products, are evident. Numerous and uniformly distributed particle aggregates are observed. While individual nano-calcium carbonate particles are difficult to resolve directly via SEM, the nucleation and uniform distribution of their hydration products can be discerned. The mechanism of NC action can be quantitatively explained by its Zeta potential: in cement pore solution (a high calcium ion environment), it typically exhibits a positive charge (Zeta potential approximately +5 to +15 mV), which facilitates its adsorption onto negatively charged initial C-S-H gel nuclei and cement particle surfaces via electrostatic attraction. Furthermore, its fine particle size enables it to fill capillary pores in the cement paste, reducing porosity, and provides numerous active nucleation sites that accelerate the nucleation and growth of hydration products (primarily C-S-H), resulting in a denser gel structure. Figure 17i displays the SEM image of the NC-2% group after 28 days. A large number of clustered, dense but loosely connected C–S–H gel agglomerates can be seen. The morphology resembles “cloud-like” or “lumpy” structures, lacking distinct crystal edges or nuclei, with wide distribution. This indicates that excessive nano-CaCO_3_ addition weakened particle dispersion, creating interfacial weak zones. Although hydration was sufficient and AFt and CH crystals were incorporated into the structure, the overall effect of reduced porosity on strength was less significant than in the 1.5 wt.% group. Figure 17j shows the SEM image of the NA-1.5% group at 28 days. The microstructure reveals well-developed hexagonal CH crystals with sharp edges and smooth crystal surfaces. These crystals were uniformly distributed, suggesting that nano-Al_2_O_3_ enhanced CH crystallization. The orderly arrangement reduced stress concentration points at the microcrack scale, thereby lowering porosity. C–S–H gels encapsulated CH crystals, indicating enhanced bonding within the matrix. The “dispersion and activation” effect of nano-Al_2_O_3_ particles improved particle distribution, effectively preventing excessive agglomeration and facilitating the co-formation of a skeleton structure with the matrix, which contributed to densification and strength.

Figure 17k shows the EDS spectrum of the NC-1% group after 28 days of curing. Two prominent Ca peaks can be observed, which are the strongest signals in the spectrum, indicating that the sample is rich in calcium. This is consistent with the incorporation of nano-CaCO_3_ and the calcium content in cement hydration products such as Ca(OH)_2_ and C–S–H. A minor peak at 0.3 keV corresponds to carbon, likely originating from unreacted CaCO_3_ particles or carbonate-type hydrates. The coexistence of C and Ca also suggests the possible presence of partially unreacted nano-CaCO_3_ grains. Figure 17l presents the EDS spectrum of the NA-1% group after 28 days. A strong Ca peak is evident, while SEM images indicate a high content of CH crystals. Although the incorporation of nano-Al_2_O_3_ increased the overall aluminum content in the system, the EDS analysis detected relatively weak Al and Si peaks. This is likely because large amounts of Al were rapidly consumed in early hydration reactions, generating amorphous C–A–H and C–A–S–H phases. These gels were uniformly distributed and formed a dense structure, leading to reduced detectable amounts of Al and Si in the EDS spectrum. This phenomenon does not imply a decrease in the overall Al or Si content, but rather reflects the indirect effect of structural densification and reaction participation.

## 4. Conclusions and Outlook

The objective of this study was to enhance the slurry performance and durability of coal gangue-based geopolymers for broader applications. Specifically, the effects of various nanomaterials (Nano-SiO_2_, GO nanosheets, Nano-CaCO_3_, and Nano-Al_2_O_3_) on the microstructural properties of geopolymers were systematically investigated, aiming to determine their effectiveness and elucidate the underlying mechanisms. The main conclusions are as follows:

1. The addition of GO nanosheets significantly reduced the fluidity of coal gangue-based geopolymers, with a 2.0 wt.% dosage leading to a flow spread decrease of 80 mm (a reduction of 73.33%). In contrast, Nano-Al_2_O_3_ had the least influence, with a reduction of only 5% at 0.5 wt.%. All four nanomaterials markedly increased the yield stress of the system, indicating that the rheological behavior of coal gangue-based geopolymers is highly sensitive to both the type and dosage of nanomaterials.
2. In the absence of nanomaterials, the strength development of the system was relatively slow, with a lower upper limit. The addition of nanomaterials altered the crack propagation and provided a certain inhibitory effect on microcracks. However, due to the relatively poor dispersibility of GO nanosheets, high dosages easily led to agglomeration. Nano-CaCO_3_ mainly acted as a filler, improving matrix compactness but exhibiting weak reactivity. Most nanomaterials showed optimal compressive strength improvements within the dosage range of 1.0–1.5 wt.%, as excessive amounts resulted in agglomeration and reduced dispersion, thereby limiting further strength enhancement.
3. Nano-SiO_2_ exhibited the strongest accelerating effect, with a dosage of 1.5 wt.% reducing the setting time most significantly; GO nanosheets also shortened the setting time but with a delayed trend; Nano-Al_2_O_3_ demonstrated a moderate yet stable accelerating effect; while Nano-CaCO_3_ mainly acted as a nucleation template, showing limited ability to accelerate setting.
4. The experimental results confirmed that porosity and water absorption of coal gangue-based geopolymers were positively correlated. With increasing nanomaterial dosage, both parameters first decreased and then increased. The lowest porosity and water absorption were achieved at dosages of 1.0 wt.% for SiO_2_, 1.0 wt.% for GO, and 1.5 wt.% for both CaCO_3_ and Al_2_O_3_.
5. SEM observations revealed that the incorporation of Nano-SiO_2_ significantly refined hydration products, yielding a denser microstructure with abundant rod- or needle-like AFt crystals that were orderly distributed. Nano-SiO_2_ also promoted pozzolanic reactions, generating more stable C-(A)-S-H gel structures and improving structural compactness. GO nanosheets provided bridging and toughening effects but suffered from dispersion issues at high dosages. Nano-Al_2_O_3_ improved early hydration kinetics and contributed to the coexistence of CH and AFt, while also enhancing the nucleation and growth of C-S-H gels. Nano-CaCO_3_ mainly acted as filler, reducing porosity but with limited reactivity. EDS analysis further confirmed that the presence of Nano-SiO_2_ and Al_2_O_3_ enhanced the Ca/Si ratio, promoted gel formation, and optimized the microstructural stability.

Based on the above conclusions, the following prospects and recommendations for future research are proposed.

1. Future work should include a systematic evaluation of the long-term durability of the materials, such as their resistance to chloride ion penetration, sulfate attack, and freeze–thaw cycles.
2. Current studies mainly focus on the influence of individual nanomaterials on the performance of coal gangue-based geopolymers. Future work should explore the synergistic effects of incorporating multiple nanomaterials. For instance, the combined use of graphene oxide and nano-SiO_2_ may yield superior improvements in strength, workability, and durability compared with single additives. Moreover, investigations should focus on determining the optimal composite ratio to mitigate agglomeration phenomena and ensure homogeneous dispersion.
3. Advanced numerical simulation methods (e.g., molecular dynamics and finite element modeling) and artificial intelligence algorithms (e.g., machine learning or deep learning models) can be employed to predict and optimize the microstructure, rheological behavior, and strength development of nanomaterial-modified geopolymers. Such approaches would enable rapid and accurate identification of optimal dosages and material combinations, thereby enhancing research efficiency and shortening the development cycle.

## Figures and Tables

**Table 1:** The chemical composition of the raw material.

Chemical Composition [%]	CaO	SiO_2_	Al_2_O_3_	Fe_2_O_3_	MgO	SO_3_	TiO_2_	K_2_O	Na_2_O	LOI
CG	1.51	63.59	23.19	5.94	0.13	0.18		3.66	0.11	1.69
OPC	59.41	22.07	5.58	3.36	1.6	2.19	1.21			4.58
SL	35.41	33.58	15.22	0.62	8.57	1.51		0.41	0.39	4.29

**Table 2:** Nanomaterial properties.

	The Number of Limited Dimensions	Purity [%]	Diameter [nm]	Diameter [mm]	Thickness [nm]	Specific Surface Area [m^2^/g]
Nano-SiO_2_	0D	99	20			200
GO nanosheets	2D	>95		<1	<100	180–280
Nano-CaCO_2_	0D	99	40			40
Nano-Al_2_O_3_	0D	99	100		Na_2_O	13–15

**Table 3:** The mix ratio of grouting materials mixed with nanomaterials.

Mixture	Water	Alkali-Solid Ratio [%]	Base Modulus	Nanoparticles [wt.%]
Reference	1.0	8%	1.7	0
NS-0.5	1.0	8%	1.7	0.5
NS-1.0	1.0	8%	1.7	1.0
NS-1.5	1.0	8%	1.7	1.5
NS-2.0	1.0	8%	1.7	2.0
GO-0.5	1.0	8%	1.7	0.5
GO-1.0	1.0	8%	1.7	1.0
GO-1.5	1.0	8%	1.7	1.5
GO-2.0	1.0	8%	1.7	2.0
NA-0.5	1.0	8%	1.7	0.5
NA-1.0	1.0	8%	1.7	1.0
NA-1.5	1.0	8%	1.7	1.5
NA-2.0	1.0	8%	1.7	2.0
NC-0.5	1.0	8%	1.7	0.5
NC-1.0	1.0	8%	1.7	1.0
NC-1.5	1.0	8%	1.7	1.5
NC-2.0	1.0	8%	1.7	2.0

**Table 4:** Rheological parameters of CGGP cement slurry.

Model	Rheological Parameters	Dosage of Nanomaterials (wt.%)								
		0.0	Nano-SiO_2_ (NS)				Graphene (GO)			
			0.5	1.0	1.5	2.0	0.5	1.0	1.5	2.0
Bingham	Yield Stress $\tau_{0}$ (Pa)	4.287	7.143	18.975	36.255	56.032	53.207	91.160	257.273	
	Plastic Viscosity $\mu_{p}$ (Pa·s)	0.021	0.040	0.034	0.035	0.055	0.088	0.087	0.052	
	R^2^	0.951	0.966	0.886	0.891	0.785	0.972	0.966	0.753	
Herschel–Bulkley	Yield Stress $\tau_{0}$ (Pa)	4.567	5.033	19.981	29.286	39.558	49.527	87.415	222.295	
	Consistency Coefficient K (Pa·s)	0.010	0.270	0.004	1.900	5.825	0.451	0.462	19.647	
	Flow Index n	1.111	0.724	1.307	0.436	0.348	0.763	0.759	0.200	
	R^2^	0.953	0.989	0.991	0.997	0.951	0.983	0.977	0.988	
**Model**	**Rheological** **Parameters**		**Nano-CaCO_3_ (NC)**				**Nano-Al_2_O_3_ (NA)**			
			**0.5**	**1.0**	**1.5**	**2.0**	**0.5**	**1.0**	**1.5**	**2.0**
Bingham	Yield Stress $\tau_{0}$ (Pa)		7.639	15.708	16.124	18.777	14.122	26.444	41.753	60.225
	Plastic Viscosity $\mu_{p}$ (Pa·s)		0.037	0.253	0.047	0.384	0.043	0.075	0.102	0.130
	R^2^		0.946	0.999	0.854	0.997	0.930	0.953	0.942	0.941
Herschel–Bulkley	Yield stress $\tau_{0}$ (Pa)		4.888	9.173	16.903	23.508	8.456	19.286	29.750	45.083
	Consistency Coefficient K (Pa·s)		0.406	1.519	0.195	0.185	1.200	1.232	2.359	2.948
	Flow Index n		0.654	0.506	0.999	1.107	0.523	0.597	0.549	0.552
	R^2^		0.983	0.942	0.999	0.999	0.994	0.997	0.998	0.998

## Data Availability

The original contributions presented in this study are included in the article. Further inquiries can be directed to the corresponding author.
